# Rare and common variants of *APOB* and *PCSK9* in Korean patients with extremely low low-density lipoprotein-cholesterol levels

**DOI:** 10.1371/journal.pone.0186446

**Published:** 2017-10-16

**Authors:** Chan Joo Lee, Yunbeom Lee, Sungha Park, Seok-Min Kang, Yangsoo Jang, Ji Hyun Lee, Sang-Hak Lee

**Affiliations:** 1 Division of Cardiology, Department of Internal Medicine, Severance Hospital, Yonsei University College of Medicine, Seoul, Korea; 2 Cardiovascular Research Institute, Yonsei University College of Medicine, Seoul, Korea; 3 Department of Medicine, Graduate School, Kyung Hee University, Seoul, Korea; 4 Department of Clinical Pharmacology and Therapeutics, College of Medicine, Kyung Hee University, Seoul, Korea; University of Padova, ITALY

## Abstract

**Background:**

Screening of variants, related to lipid metabolism in patients with extreme cholesterol levels, is a tool used to identify targets affecting cardiovascular outcomes. The aim of this study was to examine the prevalence and characteristics of rare and common variants of *APOB* and *PCSK9* in Korean patients with extremely low low-density lipoprotein-cholesterol (LDL-C) levels.

**Methods:**

Among 13,545 participants enrolled in a cardiovascular genome cohort, 22 subjects, whose LDL-C levels without lipid-lowering agents were ≤1 percentile (48 mg/dL) of Korean population, were analyzed. Two target genes, *APOB* and *PCSK9*, were sequenced by targeted next-generation sequencing. Prediction of functional effects was conducted using SIFT, PolyPhen-2, and Mutation Taster, and matched against a public database of variants.

**Results:**

Eight rare variants of the two candidate genes (five in *APOB* and three in *PCSK9*) were found in nine subjects. Two subjects had more than two different rare variants of either gene (one subject in *APOB* and another subject in *APOB/PCSK9*). Conversely, 12 common variants (nine in *APOB* and three in *PCSK9*) were discovered in 21 subjects. Among all variants, six in *APOB* and three in *PCSK9* were novel. Several variants previously reported functional, including c.C277T (p.R93C) and c.G2009A (p.G670E) of *PCSK9*, were found in our population.

**Conclusions:**

Rare variants of *APOB* or *PCSK9* were identified in nine of the 22 study patients with extremely low LDL-C levels, whereas most of them had common variants of the two genes. The common novelty of variants suggested polymorphism of the two genes among them. Our results provide rare genetic information associated with this lipid phenotype in East Asian people.

## Introduction

Individuals with hypobetalipoproteinemia have 30–40% reduction in low-density lipoprotein-cholesterol (LDL-C) levels, when they are heterozygous.[1–3] Additionally, they can show LDL-C levels <5 percentile or <50 mg/dL without lipid-lowering therapy.[4] In patients with homozygous variants, LDL-C levels are known to be much lower. Although the patients are often asymptomatic, some have increased risk for steatohepatitis.[1,2] This phenotype is associated with variants of *APOB* or P*CSK9*. Even common variants of the two genes may affect the levels of LDL-C, although this effect can be modest.[5]

In a Canadian database, 120 variants in *APOB* and 29 variants in *PCSK9*, with disease-causing potential, have been reported.[6] In affected patients, mutated *APOB* can induce truncated forms of apolipoprotein B (apoB), which are short enough to be degradated. Mutated *PCSK9* causes less degradation of LDL receptors and increases the number of these receptors on the cell, thereby reducing blood levels of LDL-C.[2] Although the reduction in LDL-C by a variant of a specific gene may not be large, its impact on the cardiovascular outcome can be greater than that of the LDL-C level assessed in adulthood.[7,8] Therefore, screening of variants related to lipid metabolism in a population with extreme LDL-C levels can be a tool to identify an important target affecting clinical outcomes.

Here, the aim of our study was to examine the prevalence and characteristics of rare and common variants of *APOB* and *PCSK9* underlying the phenotype of hypobetalipoproteinemia in Korean subjects. We used targeted next-generation sequencing, which is becoming widespread in genetic studies.[4]

## Materials and methods

### Study population

The Institutional Review Board of Severance Hospital approved the study protocols, and all subjects provided written informed consent. Subjects with extremely low levels of LDL-C were included in this study. Between November 2000 and March 2011, 13,545 subjects were enrolled in the Cardiovascular Genome Center Cohort, Yonsei University College of Medicine, Seoul, Korea. Men and women ≥18 years were recruited in this cohort when they visited Severance Hospital for cardiovascular diseases, control of risk factors, or health check-up. Participants were interviewed about their medical histories, and then underwent physical examinations. Among the total number of subjects, 22 subjects, whose LDL-C levels were ≤1 percentile (48 mg/dL) of the general Korean population, were finally analyzed. These 22 subjects were free from hypolipidemic treatment before or after enrollment to our study. This cut-off value is based on the data from 2011 Korea National Health and Nutrition Examination Survey (https://knhanes.cdc.go.kr/knhanes/eng/index.do). The level of LDL-C was assessed by direct measurement. Individuals with diagnosis of thyroid-, liver-, or kidney disease, pregnancy, cancer, or prescribed regimens that could affect lipid profiles (such as lipid-modifying agents, corticosteroids, or oral estrogen) at the time of blood sampling were excluded.

### Laboratory assessment

The levels of total cholesterol, triglyceride, high-density lipoprotein-cholesterol (HDL-C), and LDL-C were measured in all the subjects. The subjects fasted and avoided alcohol for at least 12 hours before blood sampling. Samples were analyzed within 4 hours by a laboratory that was certified by the Korean Society of Laboratory Medicine. Circulating apoB (Roche, Basel, Switzerland) and proprotein convertase subtilisin/kexin type 9 (PCSK9) levels (R&D Systems, Minneapolis, MN, USA) were measured using ELISA assays.

### Targeted sequencing and variant analysis

Two target genes were sequenced: *APOB* (MIM 107730) and *PCSK9* (MIM 607786). Genomic DNA was extracted from blood using the QiagenDNeasy kit (Qiagen, Valencia, CA, USA). For mutation analysis, a panel for targeted DNA capture and sequencing was developed by Celemics, Inc. (Seoul, Korea). Targeted sequencing and variant analysis were conducted as described. Briefly, DNA fragments, containing all coding exons and exon-intron junctions, were enriched by solution-based hybridization capture, followed by sequencing using the Illumina HiSeq 2000 platform (Illumina, Inc., San Diego, CA, USA). The quality of next-generation sequencing data including coverage information is presented in S1 Fig. Analysis of sequencing data was performed using an in-house analysis pipeline. Briefly, sequencing reads from the HiSeq 2000 raw data were sorted by index and barcode sequences. Sorted fastq files were aligned to the hg19 reference genome using the Burrows-Wheeler Aligner (BWA; ver. 0.7.12) BWA-MEM algorithm. Output SAM files were converted into BAM files and sorted using SAMtools (ver. 1.1). Duplicate removal was performed with Picard tools (ver. 1.128) MarkDuplicates. Realignment around known indel sites and Base Quality Score Recalibration (BQSR) were performed using GATK (v3.3.0) to create the final BAM files. Variants were called using the GATK v3.3.0 Unified Genotyper algorithm for loci with sequencing depth greater than or equal to 50X. Analysis of the splice regions, including sufficient intronic bases, was performed using Human Splicing Finder. Functional annotation of genetic variants was performed by ANNOVAR (ver. 2014-11-12). Functional effect predictions for single nucleotide variants were performed using SIFT, PolyPhen-2 and MutationTaster, and matched against the Korean population exome data (n = 476) and public databases of variants (dbSNP 138, Exome Variant Server and 1000 Genome project SNP [April 2012 release] from both Asian and all-population databases). We then prioritized variants according to the following criteria: 1) variants that were reported to be disease-causing in the Human Gene Mutation Database; 2) disruptive variants (nonsense, splice-site [two nucleotides on either side of the intron/exon boundary] and frameshift) that were novel or rare; and 3) novel or rare missense variants that were predicted to be deleterious by SIFT, Polyphen-2 (HumVar), or MutationTaster. Variants that met these criteria were validated by bidirectional Sanger sequencing of PCR amplicons. Databases used for identity and frequency of the variants included 1000 Genomes Project, Exome Sequencing Project 6500, and gnomAD browser (http://gnomad.broadinstitute.org/). Variants were classified as rare when minor allele frequency (MAF) <1%, whereas classified as common when MAF ≥5% in public databases.

## Results

### Clinical characteristics of study subjects

Clinical characteristics of the study subjects are described in Table 1. Mean patient age was 52 years and 64% of the patients were males; 14% of the subjects had type 2 diabetes for 0 to 27 years; the mean level of LDL-C was 39.2 mg/dL. The patients’ median apoB level was 53 mg/dL (interquartile range: 39–61 mg/dL). This median value was much lower than 90–97 mg/dL, which has been reported in prior studies in healthy Koreans.[9,10] The median PCSK9 level was 251 ng/mL (interquartile range: 190–342 ng/mL) (Table 1). The characteristics of the total cohort are shown in Table A in S1 File.

**Table 1:** Clinical characteristics of study subjects.

Variables	Study subjects (n = 22)
Age, years	52.1 ± 16.3
Male	14 (64)
Medical history	
Hypertension	12 (55)
Type 2 diabetes	3 (14)
Current smoker	7 (32)
Body mass index, kg/m^2^	23.5 ± 2.8
Laboratory values	
Total cholesterol, mg/dL	124 ± 21
TG, mg/dL	185 (96,269)
HDL-C, mg/dL	48.3 ± 17.4
LDL-C, mg/dL	39.2 ± 7.1
Non-HDL-C, mg/dL	75 ± 16
apoB, mg/dL	53 (39, 61)
PCSK9, ng/mL	251 (190, 342)

Values are presented as mean ± SD, n (%), or median (interquartile range); TG: triglyceride; HDL-C: high-density lipoprotein-cholesterol; LDL-C: low-density lipoprotein-cholesterol; apoB: apolipoprotein B; PCSK9: proprotein convertase subtilisin/kexin type 9.

### Analysis of candidate genes

**Fig 1:**
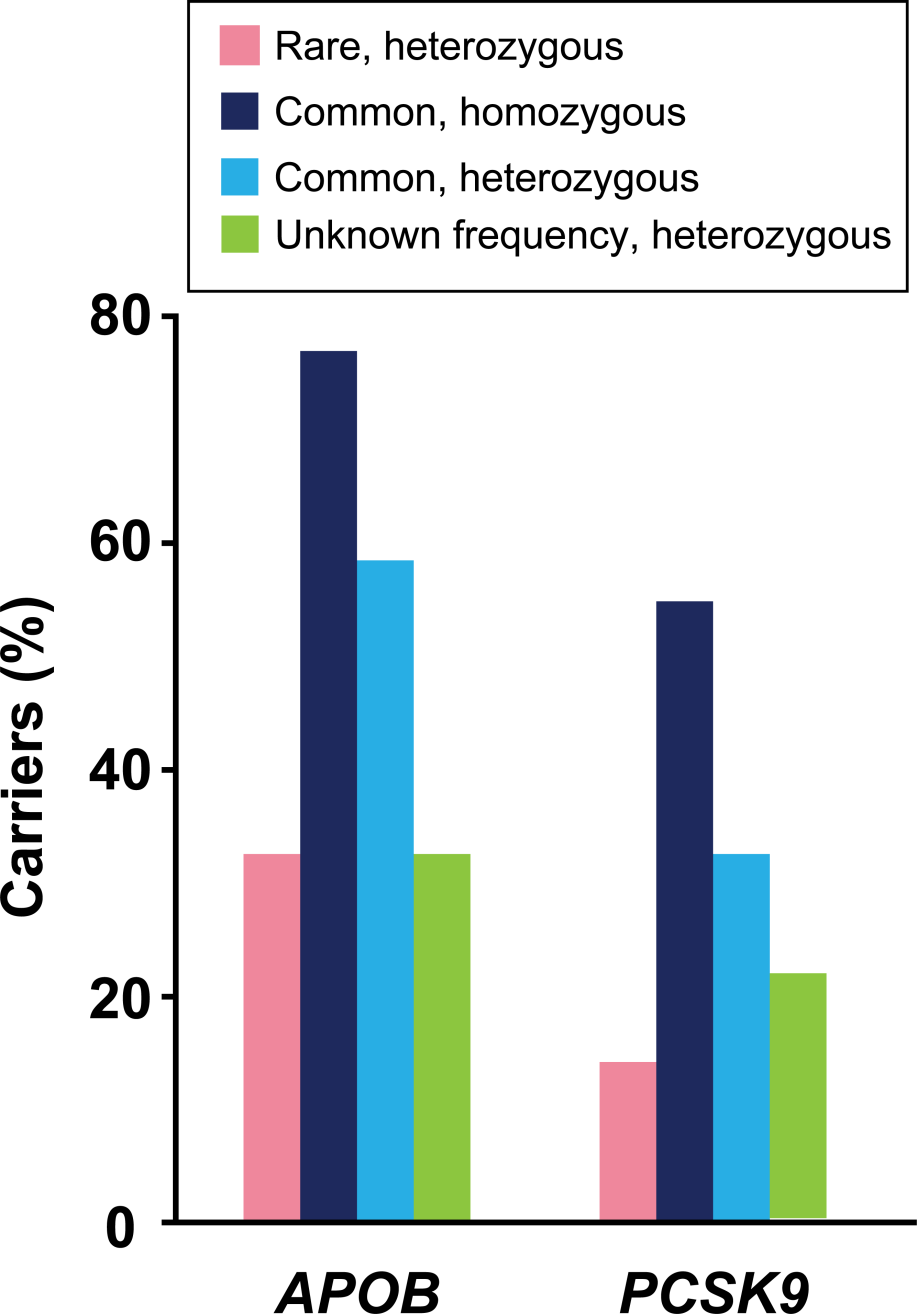
Proportion of carriers who had variants of each gene identified in 22 study subjects. With regard to *APOB*, seven subjects carried rare heterozygous variants, 17 common homozygous variants, and 14 had common heterozygous variants. Six subjects carried an *APOB* variant of unknown frequency. Conversely, with regard to *PCSK9*, three subjects carried rare heterozygous variants, 12 had common homozygous variants, and seven had common heterozygous variants. Five subjects carried *PCSK9* variants of unknown frequency.

Eight rare variants (five in *APOB* and three in *PCSK9*) of the two candidate genes were identified in nine subjects. Among all the rare variants, five were novel and five were suspected of being disease-causing (Table 2). Two subjects had more than two different rare variants in either gene (one subject in *APOB* and another subject in *APOB*/*PCSK9*). Conversely, 12 common variants (nine in *APOB* and three in *PCSK9*) of the two genes were discovered in 21 subjects. Among all the common variants, one in *APOB* was novel. Five common variants in *APOB* (e.g., c.C8216T [p.P2739L]) and two in *PCSK9* (e.g. c.G2009A [p.G670E]) were frequent and found in more than 10 individuals (Fig 1 and Tables 2 and 3). Three variants of unknown frequency (one in *APOB* and two in *PCSK9*) were identified in 10 subjects. One subject did not possess any variants of the two genes. Analysis of the splice regions revealed no variants. The type of variants in non-diabetic and diabetic subjects was analyzed and there was no significant difference therein between the two groups (Table B in S1 File).

**Table 2:** Genetic variants identified in candidate genes in study subjects.

Gene	Genomic coordinate	Nucleotide change	Mutation type	Amino acid change (rs number in dbSNP)	Allele frequency	Frequency in gnomAD Database (East Asian)	Affected patients (homo/hetero)	Report	Effect	SIFT/Polyphen/Mutation taster prediction (Clinical significance based on clinVar)
*APOB*	Rare variants									
	chr2: 21,227,212	c.G12016A	nonsynonymous SNV	p.V4006I (rs183117027)	0.045	0.006	0/2	Yes*	Unknown	Tolerated/ benign/ disease_causing (Likely benign)
	chr2: 21,228,620	c.C11120T	nonsynonymous SNV	p.A3707V (rs756381590)	0.023	<0.001	0/1	No	Unknown	Tolerated/Benign/ Polymorphism (NA)
	chr2: 21,247,843	c.C2398A	nonsynonymous SNV	p.L800M (rs183950016)	0.045	<0.001	0/2	No	Unknown	Tolerated/ possibly damaging/ disease_causing (NA)
	chr2: 21,255,236	c.G1342A	nonsynonymous SNV	p.A448T (rs752032737)	0.023	<0.001	0/1	No	Unknown	Tolerated/Benign/ polymorphism (NA)
	chr2: 21,266,783	c.T35C	nonsynonymous SNV	p.L12P (rs758450840)	0.068	<0.001	0/3	No	Unknown	Deleterious/ benign/ polymorphism (NA)
	Common variants									
	chr2: 21,225,281	c.G13013A	nonsynonymous SNV	p.S4338N (rs1042034)	0.273	0.273	1/10	Yes[11]	Unknown	Tolerated/benign/ polymorphism_ automatic (Benign/Likely benign)
	chr2: 21,231,387	c.A8353C	nonsynonymous SNV	p.N2785H (rs2163204)	0.045	0.059	0/2	Yes*	Unknown	Tolerated/Benign/ polymorphism (Conflicting interpretations of pathogenicity)
	chr2: 21,231,524	c.C8216T	nonsynonymous SNV	p.P2739L (rs676210)	0.364	0.725	3/10	Yes[11]	Familial hypercholesterolemia; hypocholesterolemia	Deleterious/probably damaging/ Possibly damaging (Benign/Likely benign)
	chr2: 21,232,803	c.A6937G	nonsynonymous SNV	p.I2313V (rs584542)	0.455	0.999	10/0	Yes*	Unknown	Tolerated/Benign/Possibly damaging (Benign)
	chr2: 21,235,475	c.A4265G	nonsynonymous SNV	p.Y1422C (rs568413)	0.545	1.000	12/0	No	Unknown	Tolerated/Benign/Possibly damaging (NA)
	chr2: 21,250,914	c.C1853T	nonsynonymous SNV	p.A618V (rs679899)	0.545	0.851	11/2	Yes[11]	Hypocholesterolemia	Tolerated/probably damaging/ Polymorphism_ automatic (Benign/Likely benign)
	chr2: 21,252,534	c.C1594T	nonsynonymous SNV	p.R532W or R505W (rs13306194)	0.159	0.135	0/6	Yes(Yilmaz)	Familial hypobetalipoproteinemia	Deleterious/probably damaging/ Disease_causing (Likely benign)
	chr2: 21,260,084	c.C581T	nonsynonymous SNV	p.T194M (rs13306198)	0.045	0.055	0/2	Yes*	Unknown	Deleterious/probably damaging/ polymorphism (Likely benign)
	chr2: 21,263,900	c.C293T	nonsynonymous SNV	p.T98I (rs1367117)	0.068	0.127	0/3	Yes[11]	Hypocholesterolemia	Tolerated/Benign/ Polymorphism automatic (Benign/Likely benign)
	Variants of unknown frequency									
	chr2: 21,266,774	c.35_44TGGCGCTGC	frameshift substitution	NA	0.136	NA	0/6	No	Unknown	
*PCSK9*	Rare variants									
	chr1: 55,505,520	c.G10A	nonsynonymous SNV	p.V4I (rs186669805)	0.023	0.002	0/1	Yes[6,12]	Hypercholesterolemia	Tolerated/Benign/B/ polymorphism (NA)
	chr1: 55,509,585	c.C277T	nonsynonymous SNV	p.R93C (rs151193009)	0.023	0.009	0/1	Yes[12,13]	Hypocholesterolemia	Tolerated/probably damaging/D/ polymorphism (Benign)
	chr1: 55,524,312	c.C1495T	nonsynonymous SNV	p.R499C (rs201395805)	0.023	<0.001	0/1	No	Unknown	Tolerated/probably damaging/ polymorphism (NA)
	Common variants									
	chr1: 55,505,668	c.C158T	nonsynonymous SNV	p.A53V (rs11583680)	0.091	0.122	0/4	Yes[13]	Unknown	Tolerated/Benign/ Polymorphism automatic (Benign/Likely benign)
	chr1: 55,524,237	c.G1420A	nonsynonymous SNV	p.V474I (rs562556)	0.409	0.993	8/2	Yes[6,14]	Hypocholesterolemia; familial hypercholesterolemia	Tolerated/Benign/ Polymorphism automatic (Benign/Likely benign)
	chr1: 55,529,187	c.G2009A	nonsynonymous SNV	p.G670E (rs505151)	0.500	0.948	10/2	Yes[6,12,13,15]	Hypocholesterolemia	Tolerated/Benign/ Polymorphism automatic (Benign/Likely benign)
	Variants of unknown frequency									
	chr1: 55,505,552	c.42_43insCTGCTGCTG	nonframeshift substitution	p.P14delinsPLLL	0.091	NA	0/4	No	Unknown	
	chr1: 55,529,225	c.2048dupA	frameshift substitution	p.H683fs	0.023	NA	0/1	No	Unknown	

SNV: single nucleotide variant, NA: not available

*: reported in genomAD browser

**Table 3:** Summary of genetic variants of target genes identified in each individual.

Patients	Sex	Age	TC (mg/dL)	TG (mg/dL)	HDL-C (mg/dL)	LDL-C (mg/dL)	Non-HDL-C (mg/dL)	Remnant-C (mg/dL)	apoB (mg/dL)	PCSK9 (ng/mL)	Numbers of variants					
											*APOB*			*PCSK9*		
											Rare	Common	Unknown	Rare	Common	Unkonwn
1	M	72	89	94	35	40	54	14	51	242	3	4	1	0	0	0
2	M	54	146	179	58	43	88	45	61	252	0	4	0	0	0	0
3	M	51	123	296	28	39	95	56	60	118	1	1	1	0	1	0
4	M	68	134	306	36	45	98	53	66	556	1	4	1	0	2	0
5	F	74	128	273	30	45	98	53	66	316	0	4	0	0	2	1
6	M	21	136	220	48	44	88	44	62	257	0	3	0	0	1	0
7	F	32	116	96	54	48	62	14	39	297	0	4	0	0	1	0
8	F	25	105	62	50	42	55	13	38	190	0	4	0	0	2	0
9	F	57	130	58	81	38	49	11	37	221	1	6	1	0	1	1
10	M	53	112	231	33	33	79	46	57	151	0	5	0	1	1	1
11	M	60	136	150	67	43	69	26	38	180	0	0	0	0	3	1
12	F	47	114	258	33	34	81	47	57	239	1	4	0	0	1	0
13	M	40	142	222	53	45	89	44	48	89	0	6	0	0	2	0
14	M	72	120	327	36	30	84	54	59	249	0	3	0	0	1	0
15	F	50	153	269	77	22	76	54	46	107	0	0	0	0	0	0
16	M	46	145	270	47	44	98	54	80	315	0	3	0	1	1	0
17	M	51	97	177	26	44	71	27	68	570	0	2	0	0	2	0
18	M	37	137	191	64	46	73	27	53	342	0	1	0	0	2	1
19	F	52	120	68	67	31	53	22	26	247	1	5	1	0	1	0
20	M	81	113	170	43	40	70	30	53	404	1	2	1	1	2	0
21	M	69	71	135	24	25	47	22	49	416	0	1	0	0	0	0
22	F	35	149	75	72	42	77	35	37	407	0	6	0	0	0	0

TC: total cholesterol; TG: triglyceride; HDL-C: high-density lipoprotein-cholesterol; LDL-C: low-density lipoprotein-cholesterol

### APOB

Five rare variants of *APOB* were discovered in seven subjects: c.G12016A (p.V4006I), c.C11120T (p.A370V), c.C2398A (p.L800M), c.G1342A (p.A448T), and c.T35C (p.L12P). All the rare variants were present in heterozygous form. One subject showed three different rare heterozygous variants of *APOB*, while one subject showed two rare heterozygous variants of *APOB* and *PCSK9*. Four of five rare variants were novel, and c.G10216A, c.G2398A, and c.T35C variants were predicted to be damaging. Meanwhile, seven common variants of this gene were found in 19 subjects. Common homozygous variants were identified in 17 subjects, whereas common heterozygous variants were shown in 14 individuals. The C8216T (p.P2739L), c.C1853T (p.A618V), c.A4265G (p.Y1422C), c.G13013A (p.S4338N), and c.A6937G (p.I2313V) variants were relatively frequent and identified in 13, 13, 12, 11, and 10 individuals, respectively. Among the common variants, c.C8216T, c.A6937G, c.A4265G, c.C1853T, c.C1594T (p.R532W), and c.C581T (p.T194M) were suspected to be disease-causing as assessed using *in silico* analysis. One variant of unknown frequency, c.35_44TGGCGCTGC was identified in six subjects (Fig 1 and Tables 2 and 3; Table C in S1 File). Circulating apoB levels did not show correlations with any specific variants in an individual.

### PCSK9

Three rare variants of *PCSK9* were found in three subjects: c.G10A (p.V4I), c.C277T (p.R93C), and c.C1495T (p.R499C). All the rare variants were heterozygous. The c.C1495T variant was novel, whereas c.C277T and c.C1495T were predicted to be damaging. Conversely, three common variants were discovered in 17 participants. Common homozygous variants were discovered in 12 subjects, while common heterozygous variants were shown in seven subjects. Among them, c.G2009A (p.G670E) and c.G1420A (p.V474I) were frequent and found in 12 and 10 individuals, respectively. The disease causality of the three common variants of *PCSK9* was not certain as assessed by *in silico* analysis. Two variants of unknown frequency were discovered in 5 individuals: c.42_43insCTGCTGCTG and c.2048dupA (p.H683fs) (Fig 1 and Tables 2 and 3; Table C in S1 File). Circulating PCSK9 levels were not associated with any specific variants in study subjects.

## Discussion

In our study population with extremely low LDL-C levels, rare variants of either *APOB* or *PCSK9* were found in nine of all subjects: seven had rare variants in *APOB*, whereas three showed rare variants in *PCSK9*. Two subjects had more than two different rare variants of either gene: one in *APOB* and one in *APOB*/*PCSK9*. Most of the study subjects had more than one common variant of the two genes: 19 had variants in *APOB* and 17 had variants in *PCSK9*. Eleven of 15 rare or common variants of *APOB* were novel, while five of six variants of *PCSK9* were known. These results provide rare and informative data about variants associated with extremely low levels of LDL-C in East Asian population.

In previous studies, the prevalence of *APOB* mutations in hypobetalipoproteinemia ranged from 44% to 64%.[4,16,17] However, genetic data for this disease in Asian patients has been scarce. The prevalence of rare variants in *APOB*, detected in our study, was 41%, which indicates a lower tendency than that in Western studies. This rate was higher than the 14% demonstrated in a Japanese study,[18] although it is difficult to compare those results with ours because of the levels of different LDL-C at enrollment. On the other hand, a considerable proportion of subjects with the phenotype did not have rare variants of the two genes. These individuals are probably influenced by the polygenic effects of lipid-related genes.

More than 60 rare variants of *APOB* have been reported in prior studies.[6,17,19] In our study, four of five rare *APOB* variants identified in the study subjects did not overlap with any of the variants reported previously. The rate of novel rare variants in *APOB* was greater than that in *PCSK9* (80% and 33%, respectively) in our results. Meanwhile, eight of nine common *APOB* variants were previously identified. Among them, the c.C1594T variant, which was recently reported in a Turkish case, is known to be of much higher minor allele frequency in East Asian population than those of other ethnicities.[20] Four other common *APOB* variants, c.C293T, c.C1853T, c.C8216T, and c.G13013A, were found in a Dutch study.[11]

The LDL-C reducing effect of c.C277T (R93C), a rare variant of *PCSK9*, has been shown in studies conducted in Japan and Canada.[12,13] We also discovered this variant in one Korean individual with this phenotype. Accordingly, the c.C277T variant may be one of the influential variants in East Asians with very low levels of LDL-C. The c.G10A (p.V4I) variant, another rare variant of *PCSK9* found in our study, was also reported in Japan and Canada.[6,12] In the study by Miyake et al, this variant was shown only in subjects with high levels of LDL-C.[12] Conversely, it did not impact the lipid profile in individuals without *LDLR* mutation,[21] and the function of this variant is not clear to date. Similar to *APOB*, diverse variants of *PCSK9* have been reported, and this gene is also considered highly polymorphic.[13,22] The c.G2009A (p.G670E) variant previously demonstrated in the United States,[15] Canada,[7,13] and Japan,[12] has shown a phenotype similar to that observed in our study. Because this variant was the most frequent among the common variants of *PCSK9* in this study, it may have considerable effect in Koreans with extremely low levels of LDL-C. Additionally, the c.C158T (p.A53V) variant, found in our study, has also been reported in a Canadian study.[13] The c.G1420A (p.V474I) variant is the second most frequent among the common variants of *PCSK9*, as observed in our study. However, it was associated with high levels of LDL-C in a Japanese study,[14] and its biological effect is incompletely proven.

Interestingly, in our analysis of the effect of allele number on body mass index, we found a positive association between the number of variant alleles in *PCSK9* and the index (r = 0.47, p = 0.03). However, there is controversy on the relationship between PCSK9 and fat accumulation.[23–25] In addition, we compared the triglyceride levels in carriers versus non-carriers of *APOB* or *PCSK9* variants and found that the levels were different with the presence of a few variants. With regard to *APOB*, the median triglyceride levels were lower in the carriers of c.C1853T (p.A618V) than in the non-carriers (174 mg/dL vs. 264 mg/dL, p = 0.04). Likewise, the levels were lower, but not significantly, in the carriers of c.T35C (p.L12P) (76 mg/dL vs. 206 mg/dL, p = 0.052) or c.C581T (p.T194M) (79 mg/dL vs. 206 mg/dL, p = 0.09) than in the non-carriers. On the contrary, the median triglyceride levels in the carriers of c.G2009A (p.G670E) in *PCSK9* tended to be higher than those in the non-carriers (227 mg/dL vs. 116 mg/dL, p = 0.06).

Our study has potential limitations. Information on the family history of the study subjects was not sufficiently available. If we could have analyzed the variants by co-segregation or functional tests, it may have provided further insight into their biological effects. In addition, many individuals showed multiple common variants in both genes, and this may cause confusion about their functionality. Although we tried to predict disease-causality of these variants by public analysis tools, we recognized that it was not perfect and was a limitation of our study. As mentioned above, variants such as c.C277T (R93C) found in our subjects and other studies are assumed to have a damaging effect on protein function. However, because the effect of most *PCSK9* variants in our study was only predicted by *in silico* analysis (Table 2), their influence on protein functionality might not be sufficiently understood in our study. We did not compare the prevalence of variants in the total cohort population and that of the study subjects. Such a comparison may have suggested an additional clinical relevance of the variants. At the same time, it was difficult to estimate per-allele LDL-C reduction effects using our data. However, we completed the main purpose of our study, characterizing the variants of the two genes in our population. Conversely, we investigated the genetic background of individuals with extremely low levels of LDL-C, and the subjects with that extreme lipid phenotype were appropriate for the aim of our study. Therefore, the number of people, who met the phenotypic criteria, could not be very large. However, the number of our study subjects was relatively large, compared with those in other studies,[4] particularly studies conducted with respect to Asian ethnicities. Finally, because of the study design and inclusion criteria, the range of LDL-C levels was quite narrow in our study. Thus, it was difficult to obtain statistical significance when we examined the association between a specific variant with the levels of LDL-C within our population. Likewise, it might be hard to find associations between circulating apoB or PCSK9 levels and specific variants in our homogenous subjects that do not have sufficient controls for comparison. Analyses, using co-segregation or comparison with total cohort population mentioned above, would be helpful for such an examination in future studies.

## Conclusion

Taken together, rare variants of either *APOB* or *PCSK9* were identified in nine of the 22 study subjects with extremely low LDL-C levels: carriers of rare variants were more frequent for *APOB* than *PCSK9*. Most of the study population had common variants in at least one of the two genes. The common novelty of variants suggested polymorphism of the two genes in this phenotype. Our results provide rare genetic information associated with extremely low levels of LDL-C in East Asian people.

## Supporting information

S1 Fig NGS data statistics of targeted sequencing. (A) Blue diamonds indicate the average sequencing depth of the target region. (B) The blue histogram represents the coverage of the target region in each sample.
(TIF)

S1 File Clinical characteristics of the total cohort and study subjects (Table A). Variants of *APOB* and *PCSK9* in non-diabetic and diabetic subjects (Table B). Genetic variants of target genes identified in each individual (Table C).
(DOCX)
