# Supplementary material for: Rare and common variants of APOB and PCSK9 in Korean patients with extremely low low-density lipoprotein-cholesterol levels
Source: PLoS One. 2017 Oct 16;12(10):e0186446. doi: 10.1371/journal.pone.0186446 (PMC5643101; doi:10.1371/journal.pone.0186446)
Supplement: S1 File — Clinical characteristics of the total cohort and study subjects (Table A). Variants of APOB and PCSK9 in non-diabetic and diabetic subjects (Table B). Genetic variants of target genes identified in each individual (Table C). (DOCX) [file pone.0186446.s002.docx]

**Supporting information**

**Table A. Clinical characteristics of the total cohort and study subjects**

| Variables | Total cohort (n=13,535) | Study subjects (n=22) |
| --- | --- | --- |
| Age, years | 60.4 ± 10.6 | 52.1 ± 16.3 |
| Male | 6,722 (50) | 14 (64) |
| Medical history |  |  |
| Hypertension | 7,234 (53) | 12 (55) |
| Type 2 diabetes | 2,293 (17) | 3 (14) |
| Current smoker | 2,004 (15) | 7 (32) |
| Body mass index, kg/m^2^ | 24.8 ± 3.1 | 23.5 ± 2.8 |
| Laboratory values, mg/dL |  |  |
| Total cholesterol | 189 ± 43 | 124 ± 21 |
| TG | 117 (84,167) | 185 (96,269) |
| HDL-C | 48.8 ± 14.7 | 48.3 ± 17.4 |
| LDL-C | 115 ± 38 | 39.2 ± 7.1 |
| Non-HDL-C | 140 ± 42 | 75 ± 16 |
| Values are presented as mean ± SD, n (), or median (interquartile range); TG: triglyceride; HDL-C: high-density lipoprotein-cholesterol; LDL-C: low-density lipoprotein-cholesterol. | | |

**Table B. Variants of APOB and PCSK9 in non-diabetic and diabetic subjects**

| Variants | Non-diabetic (n=19) | Diabetic (n=3) | P |
| --- | --- | --- | --- |
| *APOB* |  |  |  |
| c.G12016A | 2 (10.5) | 0 (0.0) | 0.999 |
| c.C2398A | 1 (5.3) | 0 (0.0) | 0.999 |
| c.C11120T | 5 (26.3) | 1 (33.3) | 0.999 |
| c.G1342A | 2 (10.5) | 0 (0.0) | 0.999 |
| c.T35C | 1 (5.3) | 0 (0.0) | 0.999 |
| c.G13013A | 10 (52.6) | 1 (33.3) | 0.999 |
| c.A8353C | 2 (10.5) | 0 (0.0) | 0.999 |
| c.C8216T | 12 (63.2) | 2 (66.7) | 0.999 |
| c.A6937G | 10 (52.6) | 1 (33.3) | 0.999 |
| c.A4265G | 10 (52.6) | 2 (66.7) | 0.999 |
| c.C1853T | 13 (68.4) | 1 (33.3) | 0.597 |
| c.C1594T | 5 (26.3) | 1 (33.3) | 0.999 |
| c.C581T | 2 (10.5) | 0 (0.0) | 0.999 |
| c.C293T | 3 (15.8) | 0 (0.0) | 0.999 |
| c.35_44TGGCGCTGC | 2 (10.5) | 0 (0.0) | 0.999 |
| *PCSK9* |  |  |  |
| c.G10A | 0 (0.0) | 1 (33.3) | 0.278 |
| c.C277T | 1 (5.3) | 0 (0.0) | 0.999 |
| c.C1495T | 1 (5.3) | 0 (0.0) | 0.999 |
| c.C158T | 4 (21.1) | 0 (0.0) | 0.942 |
| c.G1420A | 9 (47.4) | 1 (33.3) | 0.999 |
| c.G2009A | 10 (52.6) | 2 (66.7) | 0.999 |
| c.42_43insCTGCTGCTG | 4 (21.1) | 0 (0.0) | 0.942 |
| c.2048dupA | 1 (5.3) | 0 (0.0) | 0.999 |

Values are presented as n (%)

**Table C. Genetic variants of target genes identified in each individual**

| Patients | Sex | Age | TC | TG | HDL-C | LDL-C | Genes and variants: [nucleotide change], amino acid change | | | | | |
| --- | --- | --- | --- | --- | --- | --- | --- | --- | --- | --- | --- | --- |
|  |  |  |  |  |  |  | *APOB* | | | *PCSK9* | | |
| 1 | M | 72 | 89 | 94 | 35 | 40 | [c.35_44TGGCGCTGC], NA |  | # |  |  |  |
|  |  |  |  |  |  |  | **[c.T35C], p.L12P** |  |  |  |  |  |
|  |  |  |  |  |  |  | [c.C293T], p.T98I |  |  |  |  |  |
|  |  |  |  |  |  |  | [c.C1853T], p.A618V |  |  |  |  |  |
|  |  |  |  |  |  |  | **[c.C2398A], p.L800M** |  | # |  |  |  |
|  |  |  |  |  |  |  | [c.C8216T], p.P2739L |  |  |  |  |  |
|  |  |  |  |  |  |  | **[c.G12016A], p.V4006I** |  |  |  |  |  |
|  |  |  |  |  |  |  | [c.G13013A], p.S4338N |  |  |  |  |  |
| 2 | M | 54 | 146 | 179 | 58 | 43 | [c.A4265G], p.Y1422C | * | # |  |  |  |
|  |  |  |  |  |  |  | [c.A6937G], p.I2313V | * |  |  |  |  |
|  |  |  |  |  |  |  | [c.C8216T], p.P2739L |  |  |  |  |  |
|  |  |  |  |  |  |  | [c.G13013A], p.S4338N |  |  |  |  |  |
| 3 | M | 51 | 123 | 296 | 28 | 39 | [c.35_44TGGCGCTGC], NA |  | # | [c.G1420A], p.V474I | * |  |
|  |  |  |  |  |  |  | [c.A8353C], p.N2785H |  |  |  |  |  |
|  |  |  |  |  |  |  | **[c.G12016A], p.V4006I** |  |  |  |  |  |
| 4 | M | 68 | 134 | 306 | 36 | 45 | [c.35_44TGGCGCTGC], NA |  | # | [c.G1420A], p.V474I | * |  |
|  |  |  |  |  |  |  | [c.C1594T], p.R532W |  |  | [c.G2009A], p.G670E | * |  |
|  |  |  |  |  |  |  | **[c.T35C], p.L12P** |  |  |  |  |  |
|  |  |  |  |  |  |  | [c.A4265G], p.Y1422C | * | # |  |  |  |
|  |  |  |  |  |  |  | [c.C8216T], p.P2739L |  |  |  |  |  |
|  |  |  |  |  |  |  | [c.G13013A], p.S4338N |  |  |  |  |  |
| 5 | F | 74 | 128 | 273 | 30 | 45 | [c.C1853T], p.A618V | * |  | [c.42_43insCTGCTGCTG], p.P14delinsPLLL |  | # |
|  |  |  |  |  |  |  | [c.A4265G], p.Y1422C | * | # | [c.C158T], p.A53V |  | # |
|  |  |  |  |  |  |  | [c.A6937G], p.I2313V | * |  | [c.G2009A], p.G670E |  |  |
|  |  |  |  |  |  |  | [c.C8216T], p.P2739L | * |  |  |  |  |
| 6 | M | 21 | 136 | 220 | 48 | 44 | [c.C1853T], p.A618V | * |  | [c.G2009A], p.G670E | * |  |
|  |  |  |  |  |  |  | [c.A4265G], p.Y1422C | * | # |  |  |  |
|  |  |  |  |  |  |  | [c.A6937G], p.I2313V | * |  |  |  |  |
| 7 | F | 32 | 116 | 96 | 54 | 48 | [c.C581T], p.T194M |  |  | [c.G1420A], p.V474I |  |  |
|  |  |  |  |  |  |  | [c.C1853T], p.A618V | * |  |  |  |  |
|  |  |  |  |  |  |  | [c.A4265G], p.Y1422C | * | # |  |  |  |
|  |  |  |  |  |  |  | [c.C8216T], p.P2739L | * |  |  |  |  |
| 8 | F | 25 | 105 | 62 | 50 | 42 | [c.C581T], p.T194M |  |  | [c.G1420A], p.V474I |  |  |
|  |  |  |  |  |  |  | [c.C1853T], p.A618V | * |  | [c.G2009A], p.G670E | * |  |
|  |  |  |  |  |  |  | [c.A4265G], p.Y1422C | * | # |  |  |  |
|  |  |  |  |  |  |  | [c.A6937G], p.I2313V | * |  |  |  |  |
| 9 | F | 57 | 130 | 58 | 81 | 38 | [c.35_44TGGCGCTGC], NA |  | # | [c.42_43insCTGCTGCTG], p.P14delinsPLLL |  | # |
|  |  |  |  |  |  |  | **[c.T35C], p.L12P** |  |  | [c.C158T], p.A53V |  | # |
|  |  |  |  |  |  |  | [c.C1853T], p.A618V | * |  |  |  |  |
|  |  |  |  |  |  |  | [c.A4265G], p.Y1422C | * | # |  |  |  |
|  |  |  |  |  |  |  | [c.A6937G], p.I2313V | * |  |  |  |  |
|  |  |  |  |  |  |  | [c.C8216T], p.P2739L |  |  |  |  |  |
|  |  |  |  |  |  |  | [c.A8353C], p.N2785H |  |  |  |  |  |
|  |  |  |  |  |  |  | [c.G13013A], p.S4338N |  |  |  |  |  |
| 10 | M | 53 | 112 | 231 | 33 | 33 | [c.C1594T], p.R532W |  |  | **[c.C277T], p.R93C** |  |  |
|  |  |  |  |  |  |  | [c.C1853T], p.A618V | * |  | [c.G2009A], p.G670E | * |  |
|  |  |  |  |  |  |  | [c.A4265G], p.Y1422C | * | # | [c.2048dupA], p.H683fs |  | # |
|  |  |  |  |  |  |  | [c.C8216T], p.P2739L |  |  |  |  |  |
|  |  |  |  |  |  |  | [c.G13013A], p.S4338N |  |  |  |  |  |
| 11 | M | 60 | 136 | 150 | 67 | 43 |  |  |  | [c.42_43insCTGCTGCTG], p.P14delinsPLLL |  | # |
|  |  |  |  |  |  |  |  |  |  | [c.C158T], p.A53V |  | # |
|  |  |  |  |  |  |  |  |  |  | [c.G1420A], p.V474I |  |  |
|  |  |  |  |  |  |  |  |  |  | [c.G2009A], p.G670E | * |  |
| 12 | F | 47 | 114 | 258 | 33 | 34 | **[c.G1342A], p.A448T** |  | # | [c.G2009A], p.G670E | * |  |
|  |  |  |  |  |  |  | [c.C1594T], p.R532W |  |  |  |  |  |
|  |  |  |  |  |  |  | [c.A4265G], p.Y1422C | * | # |  |  |  |
|  |  |  |  |  |  |  | [c.C8216T], p.P2739L |  |  |  |  |  |
|  |  |  |  |  |  |  | [c.G13013A], p.S4338N |  |  |  |  |  |
| 13 | M | 40 | 142 | 222 | 53 | 45 | [c.C1594T], p.R532W |  |  | [c.G1420A], p.V474I | * |  |
|  |  |  |  |  |  |  | [c.C1853T], p.A618V | * |  | [c.G2009A], p.G670E | * |  |
|  |  |  |  |  |  |  | [c.A4265G], p.Y1422C | * | # |  |  |  |
|  |  |  |  |  |  |  | [c.A6937G], p.I2313V | * |  |  |  |  |
|  |  |  |  |  |  |  | [c.C8216T], p.P2739L |  |  |  |  |  |
|  |  |  |  |  |  |  | [c.G13013A], p.S4338N |  |  |  |  |  |
| 14 | M | 72 | 120 | 327 | 36 | 30 | [c.C1594T], p.R532W |  |  | [c.G2009A], p.G670E |  |  |
|  |  |  |  |  |  |  | [c.C8216T], p.P2739L |  |  |  |  |  |
|  |  |  |  |  |  |  | [c.G13013A], p.S4338N |  |  |  |  |  |
| 15 | F | 50 | 153 | 269 | 77 | 22 |  |  |  |  |  |  |
| 16 | M | 46 | 145 | 270 | 47 | 44 | [c.C1853T], p.A618V | * |  | **[c.G10A], p.V4I** |  |  |
|  |  |  |  |  |  |  | [c.A4265G], p.Y1422C | * | # | [c.G2009A], p.G670E | * |  |
|  |  |  |  |  |  |  | [c.A6937G], p.I2313V | * |  |  |  |  |
| 17 | M | 51 | 97 | 177 | 26 | 44 | [c.C1853T], p.A618V | * |  | [c.G1420A], p.V474I | * |  |
|  |  |  |  |  |  |  | [c.A6937G], p.I2313V | * |  | [c.G2009A], p.G670E | * |  |
| 18 | M | 37 | 137 | 191 | 64 | 46 | [c.C1853T], p.A618V | * |  | [c.42_43insCTGCTGCTG], p.P14delinsPLLL |  | # |
|  |  |  |  |  |  |  |  |  |  | [c.C158T], p.A53V |  | # |
|  |  |  |  |  |  |  |  |  |  | [c.G1420A], p.V474I |  |  |
| 19 | F | 52 | 120 | 68 | 67 | 31 | [c.35_44TGGCGCTGC], NA |  | # | [c.G1420A], p.V474I | * |  |
|  |  |  |  |  |  |  | [c.C293T], p.T98I |  |  |  |  |  |
|  |  |  |  |  |  |  | [c.C1853T], p.A618V |  |  |  |  |  |
|  |  |  |  |  |  |  | [c.A6937G], p.I2313V | * |  |  |  |  |
|  |  |  |  |  |  |  | [c.C8216T], p.P2739L |  |  |  |  |  |
|  |  |  |  |  |  |  | **[c.C11120T], p.A3707V** |  | # |  |  |  |
|  |  |  |  |  |  |  | [c.G13013A], p.S4338N |  |  |  |  |  |
| 20 | M | 81 | 113 | 170 | 43 | 40 | [c.35_44TGGCGCTGC], NA |  | # | [c.G1420A], p.V474I | * |  |
|  |  |  |  |  |  |  | [c.C293T], p.T98I |  |  | **[c.C1495T], p.R499C** |  | # |
|  |  |  |  |  |  |  | **[c.C2398A], p.L800M** |  | # | [c.G2009A], p.G670E | * |  |
|  |  |  |  |  |  |  | [c.G13013A], p.S4338N | * |  |  |  |  |
| 21 | M | 69 | 71 | 135 | 24 | 25 | [c.C8216T], p.P2739L | * |  |  |  |  |
| 22 | F | 35 | 149 | 75 | 72 | 42 | [c.C1594T], p.R532W |  |  |  |  |  |
|  |  |  |  |  |  |  | [c.C1853T], p.A618V | * |  |  |  |  |
|  |  |  |  |  |  |  | [c.A4265G], p.Y1422C | * | # |  |  |  |
|  |  |  |  |  |  |  | [c.A6937G], p.I2313V | * |  |  |  |  |
|  |  |  |  |  |  |  | [c.C8216T], p.P2739L |  |  |  |  |  |
|  |  |  |  |  |  |  | [c.G13013A], p.S4338N |  |  |  |  |  |
| TC: total cholesterol; TG: triglyceride; HDL-C: high-density lipoprotein-cholesterol; LDL-C: low-density lipoprotein-cholesterol; rare variants in bold character; variants with unknown frequency underlined; *: homozygous; #: novel variants; NA: not available | | | | | | | | | | | | |
